# Supplementary figures and images for: lncRNA MALAT1 mediates osteogenic differentiation of bone mesenchymal stem cells by sponging miR-129-5p
Source: PeerJ. 2022 Apr 22;10:e13355. doi: 10.7717/peerj.13355 (PMC9037136; doi:10.7717/peerj.13355)

A

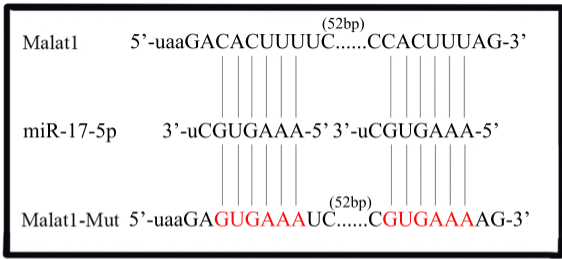

B

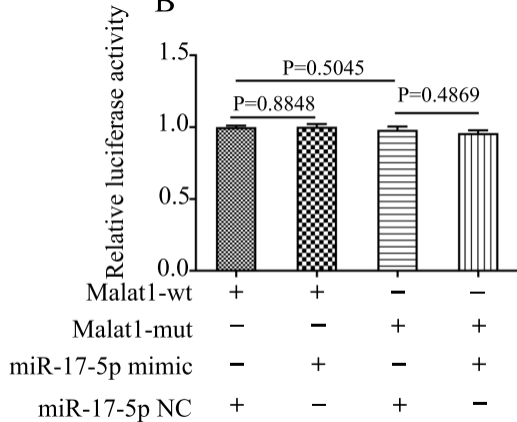

Supplement: Supplemental Information 6 — (A) Complementary bases between the sequences are labeled with red font. The sequence of the mutant lncRNA Malat1 construct is also shown as underlined. (B) Dual-luciferase reporter assay of 293T cells co-transfected with lncRNA Malat1, or lncRNA Malat1-Mut and with miR-17-5p mimic or miR-NC. [file peerj-10-13355-s006.pdf]

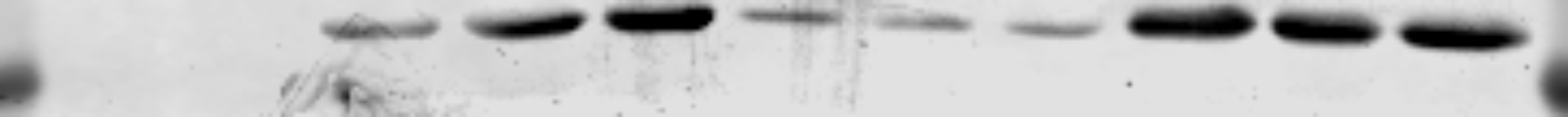

Supplement: Supplemental Information 7 [file peerj-10-13355-s007.zip › Uncropped blots/Figure10.WB-inhibitor-GAPDH(20201101).tif]

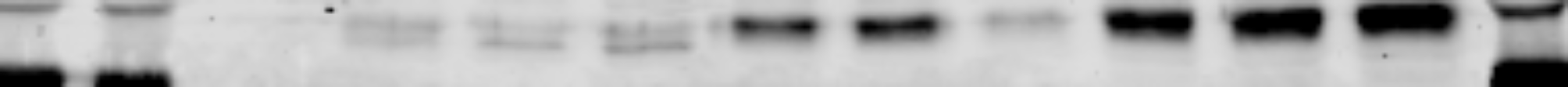

Supplement: Supplemental Information 7 [file peerj-10-13355-s007.zip › Uncropped blots/Figure10.WB-inhibitor-RUNX2(20201101).tif]

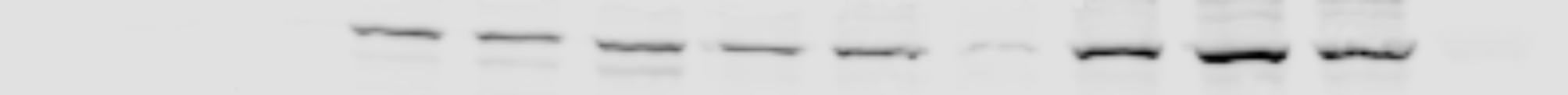

Supplement: Supplemental Information 7 [file peerj-10-13355-s007.zip › Uncropped blots/Figure10.WB-inhibitor-STAT1(20201101).tif]

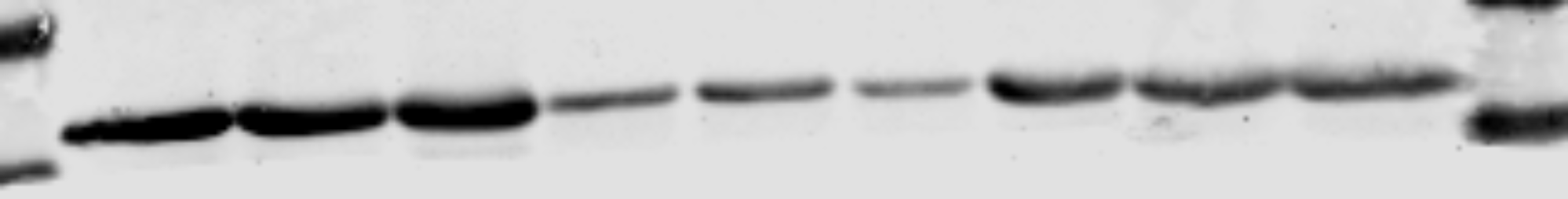

Supplement: Supplemental Information 7 [file peerj-10-13355-s007.zip › Uncropped blots/Figure11.WB-inhibitor-gapdh(20201109).tif]

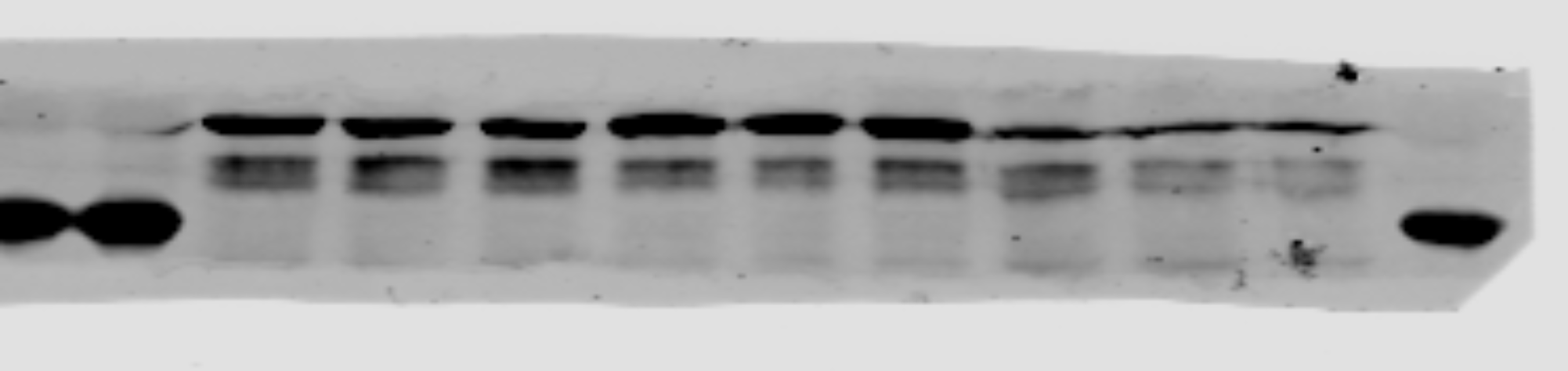

Supplement: Supplemental Information 7 [file peerj-10-13355-s007.zip › Uncropped blots/Figure11.WB-inhibitor-RUNX2-laminb(20201109).tif]

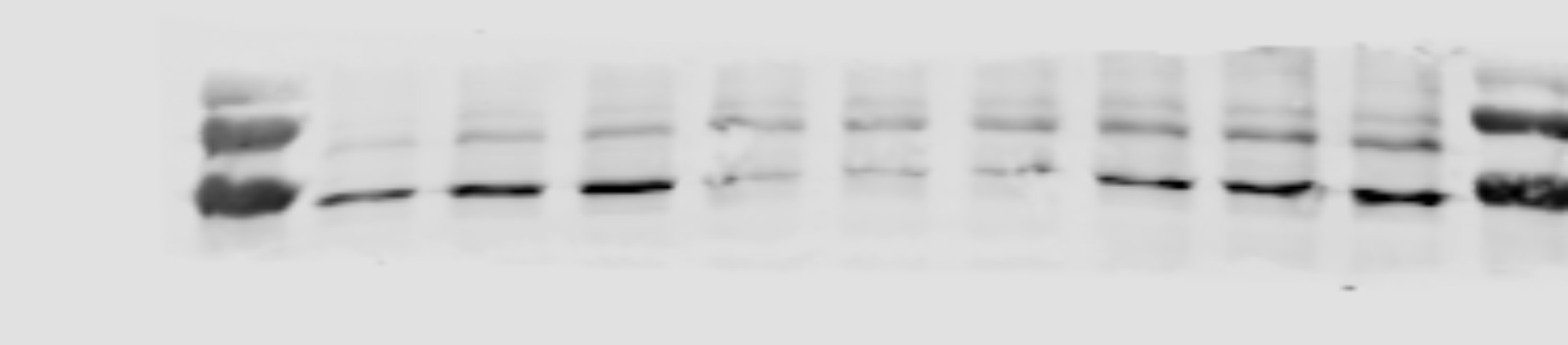

Supplement: Supplemental Information 7 [file peerj-10-13355-s007.zip › Uncropped blots/Figure11.WB-inhibitor-Stat1(20201109).tif]

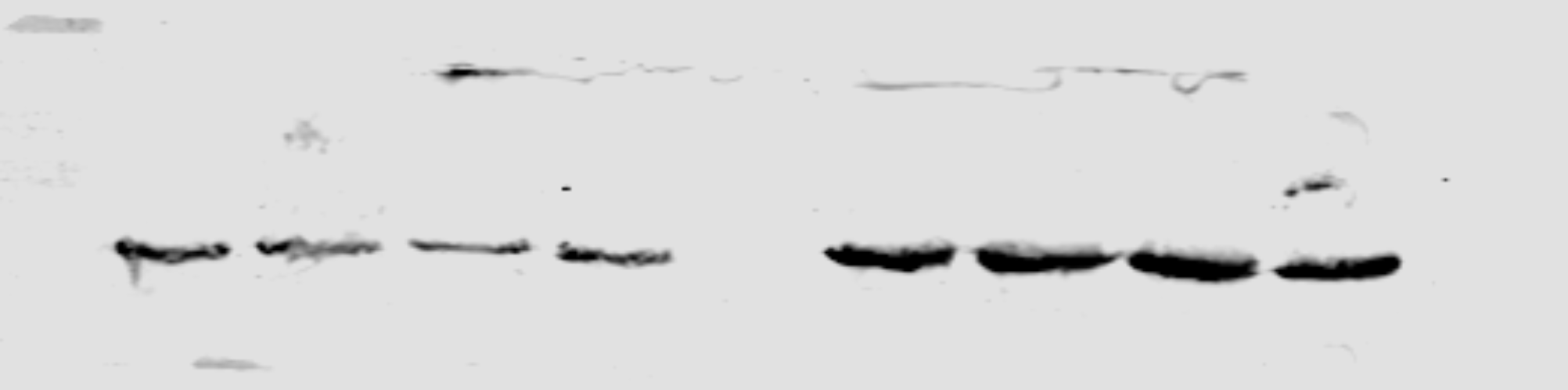

Supplement: Supplemental Information 7 [file peerj-10-13355-s007.zip › Uncropped blots/Figure13.WB-mimic-gapdh(20200902).tif]

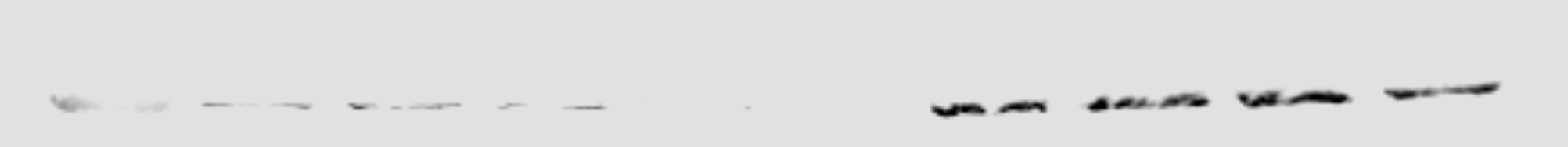

Supplement: Supplemental Information 7 [file peerj-10-13355-s007.zip › Uncropped blots/Figure13.WB-mimic-STAT1(20200902).tif]

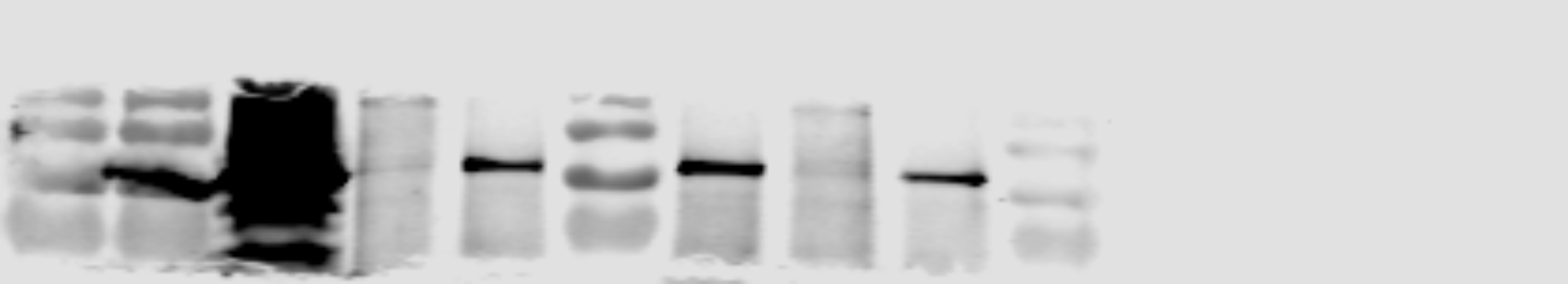

Supplement: Supplemental Information 7 [file peerj-10-13355-s007.zip › Uncropped blots/Figure16.WB-AGO(20201222).tif]

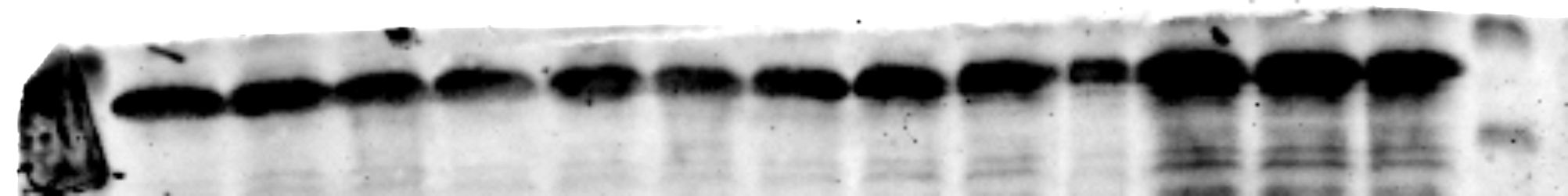

Supplement: Supplemental Information 7 [file peerj-10-13355-s007.zip › Uncropped blots/Figure8. WB-gapdh(20201026).tif]

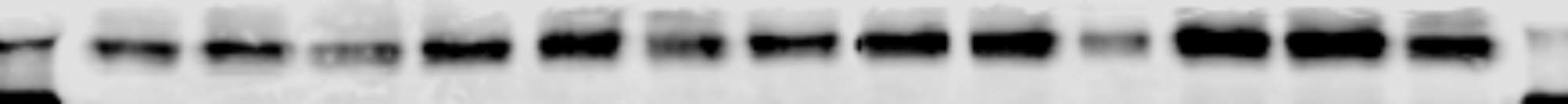

Supplement: Supplemental Information 7 [file peerj-10-13355-s007.zip › Uncropped blots/Figure8.WB-runx2(20201026).tif]

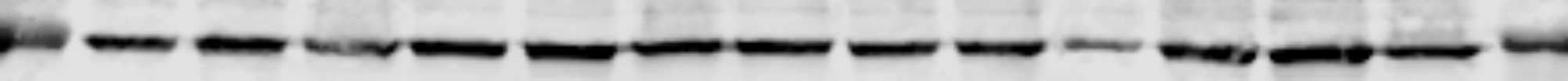

Supplement: Supplemental Information 7 [file peerj-10-13355-s007.zip › Uncropped blots/Figure8.WB-stat1(20201026).tif]

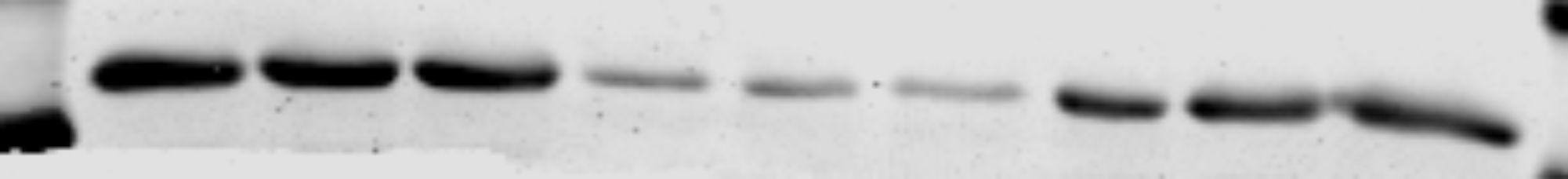

Supplement: Supplemental Information 7 [file peerj-10-13355-s007.zip › Uncropped blots/Figure9.WB-ASO-GAPDH(20201103).tif]

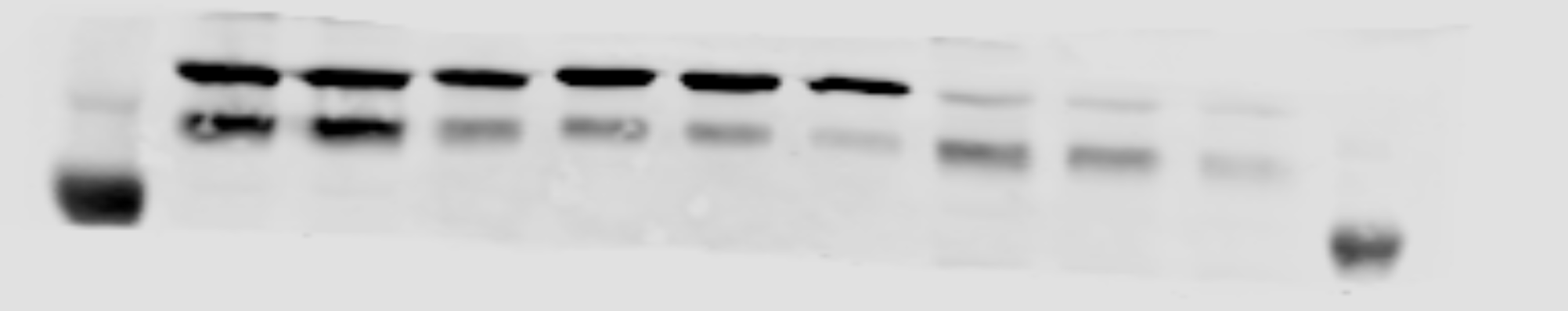

Supplement: Supplemental Information 7 [file peerj-10-13355-s007.zip › Uncropped blots/Figure9.WB-ASO-laminb(20201103).tif]

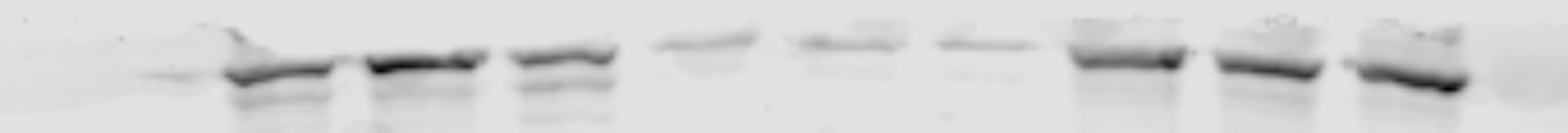

Supplement: Supplemental Information 7 [file peerj-10-13355-s007.zip › Uncropped blots/Figure9.WB-ASO-STAT1(20201103).tif]
